# A new approach to osteoarthritis: gut microbiota

**DOI:** 10.1590/1806-9282.20241528

**Published:** 2025-05-02

**Authors:** Mustafa Aydin, Gulcin Alp Avci, Ulku Irem Yilmaz, Emre Avci

**Affiliations:** 1 University of Health Sciences, Gulhane Faculty of Medicine, Department of Orthopedics and Traumatology – Ankara, Türkiye.; 2 University of Health Sciences, Gulhane Faculty of Dentistry, Department of Basic Medical Sciences – Ankara, Türkiye.; 3 University of Health Sciences, Gulhane Vocational School of Health, Department of Pathology – Ankara, Türkiye.; 4 University of Health Sciences, Gulhane Faculty of Pharmacy, Department of Biochemistry – Ankara, Türkiye.

**Keywords:** Knee osteoarthritis, Microbiota, Intestinal barrier permeability

## Abstract

**OBJECTIVE:**

Studies investigating the relationship between the gut microbiome and osteoarthritis have increased in recent years. However, data on the relationship between joints and the gut microbiome are limited. The aim of this study was to determine whether there is a relation between knee joint fluid and gut microbiota in patients with knee osteoarthritis.

**METHODS:**

This study included 40 individuals, 20 of whom were diagnosed with knee osteoarthritis and 20 of whom were considered healthy controls. Joint-fluid and stool samples were taken from the participants. Bacteria isolated from the samples were identified using a matrix-assisted laser desorption ionization-time of flight-mass spectrometry device.

**RESULTS:**

Twenty-nine different bacteria were isolated from the stool samples and five bacteria were isolated from the joint-fluid samples. In our study, the same types of microorganisms (*Enterococcus faecium* and *Staphylococcus hominis*) were isolated from the stool and joint-fluid samples.

**CONCLUSION:**

The data obtained in our study shed light on the uncertainty of how microorganisms, especially those identified in the knee and hip in the literature, reach these regions. The presence of intestinal bacteria in the knee joint fluid of osteoarthritis patients indicates that intestinal bacteria, especially in individuals with a weak immune system, malnutrition, and obesity, pass through the intestinal wall and reach other parts of the body via the bloodstream, a condition also known as "leaky gut."

## INTRODUCTION

Knee osteoarthritis (OA) is a chronic joint disease characterized by bone hyperplasia and inflammatory destruction^1^. While it usually manifests itself with pain, it can also show symptoms, such as limitation of movement, stiffness, swelling, locking, and numbness. Hip and knee OA, estimated to affect approximately 300 million people worldwide, develops under the influence of systemic, local, and genetic factors^2,3^. In addition to articular cartilage, OA can also affect other structures associated with the joint, such as subchondral bone, adjacent connective tissue, and synovial membrane. Chondrocyte cells, which form the structure of the cartilage, are immobile and cannot renew themselves; therefore, chondrocyte death has an important role in the pathogenesis of OA^4,5^.

The gut microbiome is a system in which trillions of symbiotic bacteria colonize our body and is of vital importance for our health. These microorganisms play an active role in various biological processes, such as metabolism, immune system, and neurological functions^5,6^. Gut microbes play a critical role in maintaining metabolic balance, development of the immune system, building resistance to infections, and production of neurotransmitters. Imbalances in this microbiota can lead to serious health problems, such as obesity, diabetes, metabolic diseases, and cancer. When used in appropriate amounts, probiotic supplements provide significant benefits to host health^7^. Additionally, the gut microbiome is a source of important vitamins and helps maintain metabolic balance, immune system development, and neurotransmitter production. Within this microbial community, Firmicutes and Bacteroidetes are the most common groups, but other bacterial groups are also present^8,9^.

It is observed that the intestinal microbiota may contribute to the pathogenesis of joint diseases, especially OA, by affecting bone metabolism. This can be explained as the intestinal microbiota may affect the etiopathology of OA at both systemic and local levels, contributing to the development of this disease by paving the way for the onset of immune-metabolic disorders^9^. Additionally, it should be noted that the intestinal microbiota may accelerate the progression of inflammation-related diseases and affect the pathophysiology of OA. More research is needed on how OA risk factors such as aging, dietary habits, and obesity affect the gut microbiota. It is thought that the gut–bone relationship may be a promising target in the prevention and treatment of OA^9-11^. Recent studies have provided concrete evidence of this connection, and to fully explain these mechanisms, the role of gut microbiome-derived immune-metabolic disorders in the pathogenesis of OA needs to be further investigated^12-15^. It is important to conduct new research to further understand the effects of gut microbiomes on health. A more in-depth study on the role of these microorganisms in metabolism, immune system, and neurological functions may help develop more effective strategies for the management of health problems^16^. Additionally, collecting more data on the complexity of the gut microbiome and the contribution of different groups of bacteria will help enrich scientific research in this field and develop new approaches for disease diagnosis and treatment. Our study aimed to determine whether there is a relationship between joints and gut microbiota in individuals with knee OA.

## METHODS

### Patient population

Patients with stage I, II, III, and IV knee OA, who were diagnosed according to the Kellgren-Lawrence system and the criteria set by the ACR, and who applied to the Department of Orthopedics and Traumatology between June and July 2024 were included in the study. This study was approved by the Clinical Research Ethics Committee of Gulhane Training and Research Hospital (decision no. 2024/264, dated: 28.05.2024), and all the study subjects participated voluntarily. The study was conducted in accordance with the principles of the Declaration of Helsinki.

The sample size of individuals participating in the research was evaluated with G-power analysis. In this context, a total of 40 individuals were included in the study, 20 of whom were diagnosed with knee OA and 20 of whom were considered healthy controls.

In our outpatient clinic, joint fluids taken from patients diagnosed with knee OA during treatment and about to be discarded were placed in sterile Falcon tubes and stored at −20°C until the study was performed. To investigate clinical findings during diagnosis and treatment, routine stool examinations were requested from patients with gastrointestinal system complaints according to the anamnesis, and the stool samples of these patients were stored for use in microbiota analyses.

### Bacterial culture

Stool samples taken from patients were diluted with phosphate-buffered saline (PBS) at concentrations of 10^1^ and 10^5^ before the study. The joint-fluid and diluted stool samples were spread on eosin-methylene blue (EMB), plate count agar (PCA), and de Man–Rogosa–Sharpe (MRS) agar from 10^4^ and 10^5^ dilutions. The plates of the stool samples were incubated in a 37°C oven for 16–24 h. The plates of the joint-fluid samples were incubated for 16–24 h in a 37°C oven with 5% CO_2_.

The resulting bacterial colonies were analyzed both macroscopically and microscopically. Colony counts were made on the plates with growth after incubation, and the first stage of the analysis was carried out by evaluating the morphological characteristics of the colonies. Then, bacterial colonies with different morphologies were typed using microbiological staining techniques (Gram staining) and biochemically (catalase test, coagulase test, motility test, oxidase test, etc.). The bacteria isolated from the samples were identified by a matrix-assisted laser desorption ionization-time of flight-mass spectrometry device, and antimicrobial susceptibility examination was performed by the disk–diffusion method. A score of 2.0 or higher indicates high reliability at the species level, and a score of 1.7–2.0 indicates a match at the genus level^17^.

### Statistical analysis

Before the study, the sample size of individuals participating in the research was evaluated via G*Power analysis. The "Statistical Package for the Social Sciences" (SPSS) program version 22.0 (IBM Corp., Armonk, NY, USA) was used to evaluate the data obtained in the study. The data to normal distribution were examined by the Kolmogorov-Smirnov test. Parametric tests were used for data with normal distribution according to the Kolmogorov-Smirnov test result.. A value of p<0.05 was accepted as the level of statistical significance.

## RESULTS

**Figure 1:**
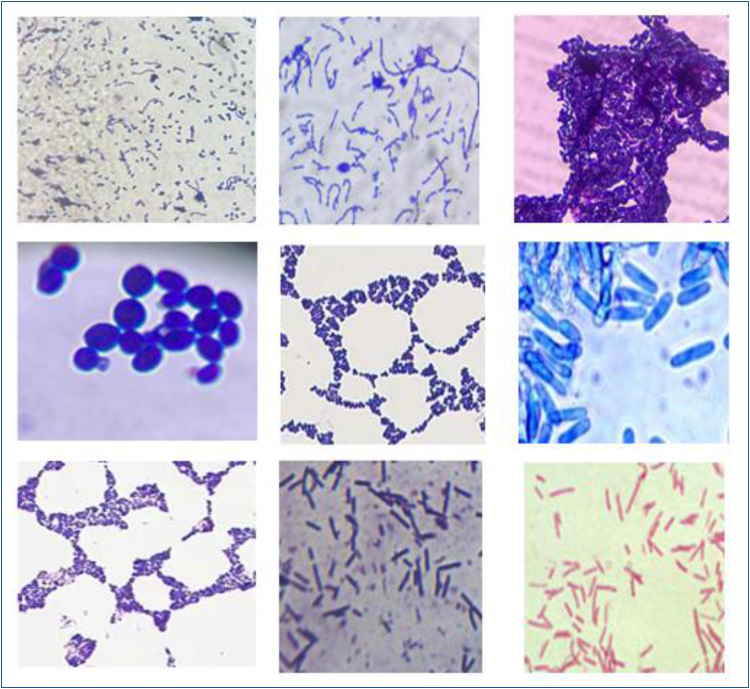
The light microscope images of some microorganisms from stool and joint-fluid samples (100×).

A total of 40 OA patients (24 females and 16 males) were included in this study. The age range of the patients was 51–73 years, with a mean age of 61.6±8.0 years. According to the obtained data, the number of samples from which bacteria were isolated (18 Females/8 Males) and from which they were not also evaluated according to gender (Figure 1). As a result of the analysis performed with these data using the "chi-square test" in the SPSS program, Pearson's value was calculated as 0.685. Based on this result, with a value of p>0.05, it was reported that there was no significant difference between the number of bacteria isolated from the samples taken from the OA patients included in the study and patient gender.

A total of 29 different types of bacteria were isolated from the stool samples. It was determined that the number of pathogenic and opportunistic pathogenic microorganisms increased in the intestinal flora of these patients. Also, another five types of bacteria were isolated from the stool samples. The obtained data are shown in Table 1. In our study, the same species were found in both the stool and joint-fluid samples. The most notable of these are *Enterococcus faecium* and *Staphylococcus hominis*. Patients were directed to treatment in the clinic after opportunistic pathogen bacteria were identified in the joint fluid. While some of the species detected in the stool were members of the normal flora, the presence of species found through food was also detected.

**Table 1:** Microorganisms from stool and joint-fluid samples.

Microorganisms from the stool sample	%	Microorganisms from the joint-fluid sample	%
*Enterococcus faecium*	8	*Streptomyces lavendulae*	20
*Enterococcus mundtii*	2	*Myroides odoratus/odoratimimus*	20
*Enterococcus durans*	2	*Colletotrichum gloeosporioides*	10
*Candida albicans*	3	*Enterococcus faecium*	20
*Lactobacillus acidophilus*	9	*Staphylococcus hominis*	30
*Lacticaseibacillus paracasei*	9		
*Limosilactobacillus reuteri*	10		
*Lactobacillus gasseri*	9		
*Levilactobacillus brevis*	12		
*Staphylococcus hominis*	10		
*Stenotrophomonas maltophilia*	1		
*Escherichia coli*	8		
*Enterobacter hormaechei*	1		
*Enterobacter ludwigii*	1		
*Enterobacter asburiae*	1		
*Klebsiella pneumoniae*	1		
*Micrococcus luteus*	1		
*Pseudocitrobacter polychromogenes*	1		
*Pseudomonas putida*	1		
*Kluyveromyces marxianus*	1		
*Geodermatophilus bullaregiensis*	1		
*Microbacterium maritypicum*	1		
*Micrococcus luteus*	1		
*Peribacillus muralis*	1		
*Peribacillus simplex*	1		
*Secundilactobacillus malefermentans*	1		
*Kluyveromyces ascorbata*	1		
*Flavobacterium columnare*	1		
*Odoribacter splanchnicus*	1		

Among the isolated bacteria from the joint-fluid samples, bacteria thought to be biotechnologically effective, especially in the health field, were also identified. One of these is *Streptomyces lavendulae*. *Myroides odoratus/odoratimimus* detected in the joint fluid is generally found in soil and water, but it can be pathogenic in those with underlying diseases, especially in immunosuppressed patients. Species detected in the stool samples are generally members of the normal flora (*E. faecium*, *Lactobacillus acidophilus*, and *Levilactobacillus brevis*) or species transmitted through food.

## DISCUSSION

In the study of joint pain, the investigation of the connection between the musculoskeletal system and the intestinal system may be an interesting topic. Literature information suggests that intestinal permeability which plays an important role in many diseases may create a new diagnostic and treatment protocol in this regard. With time, individual intestinal health is increasingly becoming crucial in chronic diseases^18^.

In recent years, in addition to genetic predisposition, physical inactivity, and nutritional disorders leading to obesity and metabolic syndrome, OA has become more common. The literature shows that all these conditions are closely related to the intestinal microbiota. Systemic and local inflammation plays an important role in the pathogenesis of OA. It has been reported in the literature that deteriorated cartilage may lead to the formation of inflammatory cytokines and metalloproteases^19,20^. Many studies have found several immune cells, including B cells, T cells, lymphoid follicles, granulocytes, and plasma cells, in the synovium of OA patients and have suggested that the innate/adaptive immune response has a central effect on the pathogenesis of OA^21^. To date, researchers have identified various risk factors for OA, such as age, gender, nutrition, obesity and metabolic syndrome, genetic background, inflammation, and intestinal microbiome^19,22^. However, pain relief or joint replacement is usually applied to OA patients. However, considering the studies and findings obtained, determining the treatment target of the disease is very important.

In our study, many normal flora members such as *E. faecium*, *L. acidophilus*, and *L. brevis* were detected, especially in stool samples. In addition to these, food pathogens and opportunistic pathogens were also observed to have a place in the flora. *Enterococci*, which are found in the natural flora of the intestine, oral cavity, and vagina, are known to be mostly avirulent in healthy individuals, but they often behave as pathogens in hospitalized patients^23,24^. *Kluyvera* is a relatively newly identified member of the Enterobacteriaceae family and rarely causes infection in humans^25^. In our study, it was detected in only one patient. *Odoribacter splanchnicus* is a Gram-negative anaerobic bacterium normally found in the intestines, known for its tumor-suppressive and immunomodulatory activities. It is an extremely rare pathogen of human infection, mainly reported with bacteremia infection^26,27^. Only a few cases of human infection have been reported, and it was detected in only one patient in our study. Some species, especially known as fish pathogens, can temporarily colonize the human intestinal system. Similar microorganisms (*Micrococcus luteus* and *Flavobacterium columnare*) were also detected in our study.

*S. lavendulae* produces mitomycin C (MC), and mitomycin is an important biotechnological agent used in anticancer therapy. *S. lavendulae* also produces complestatins and protease inhibitors with antiviral activities^19^. The other agent identified, *M. odoratimimus*, is an uncommon opportunistic pathogen, although it has been reported in the literature to be isolated from various bodily fluids. Since it is widely found in the environment, infections encountered may occur after contact with contaminated water^28^. *Colletotrichum* species are common pathogens, especially for plant anthracnose, but have recently been reported in the literature as opportunistic human pathogens causing keratitis and subcutaneous fungal infections, potentially leading to life-threatening systemic dissemination^29^.

### Strengths and limitations

Our study was limited in terms of the data obtained because it was conducted on OA patients only, and the small sample size of our study and the inclusion of only those who applied to the hospital constitute important limitations. However, it is an important study to determine the microbial situation in OA patients. Nevertheless, further studies on the subject and the inclusion of more patients will also increase statistical power.

## CONCLUSION

Our study has drawn attention to the relationship between joint diseases and microbiota. The most important innovation that our study has added to the literature is the demonstration of intestinal flora bacteria in the joint fluids of patients with leaky gut syndrome. In addition, the imbalance in the intestinal flora in OA patients has been revealed and findings that will support the treatment of these patients have been reached.

The data obtained in our study shed light on the uncertainty of how microorganisms, especially those identified in the knee and hip in the literature, reach these regions. The presence of intestinal bacteria in the knee joint fluid of OA patients indicates that intestinal bacteria, especially in individuals with a weak immune system, pass through the intestinal wall and reach other parts of the body via the bloodstream, a condition also known as "leaky gut."
